# A novel physiological significance of natriuretic peptides in cancer metastasis

**DOI:** 10.1186/2050-6511-14-S1-O34

**Published:** 2013-08-29

**Authors:** Kenji Kangawa, Takashi Nojiri, Hiroshi Hosoda, Takeshi Tokudome, Meinoshin Okumura

**Affiliations:** 1 Department of Biochemistry, National Cerebral and Cardiovascular Center Research Institute, Suita, Osaka, Japan; 2 Department of General Thoracic Surgery, Osaka University Graduate School of Medicine, Suita, Osaka, Japan

## Background

Cancer metastasis is the most common cause of death in cancer patients. Although surgical resection remains the only potentially curative treatment for lung cancer, lung cancer patients after curative surgery still have high risk of cancer recurrence. If we can perform any prophylactic strategy during the perioperative period for cancer recurrence, surgical outcome for lung cancer could be improved significantly.

Atrial natriuretic peptide (ANP) has been used clinically for the treatment with heart failure in Japan, and exhibits a wide range of biological activities including beneficial effects on cardiovascular system through binding the guanylate cyclase-A (GC-A) receptor. We previously reported that ANP administration during the perioperative period had a prophylactic effect on postoperative cardiopulmonary complications in lung cancer patients. We performed the follow-up survey in these patients, and found that the incidence of cancer recurrence after curative surgery for lung cancer was significantly lower in the ANP-treated patients compared to the control patients (surgery alone). The objective of the present study was to investigate the mechanism of ANP in the prevention of cancer metastasis.

## Methods

We used B16 mice melanoma cell line, which don't have the GC-A receptor, in the experimental hematogenous metastasis model in mice. ANP infusion (subcutaneously via osmotic-pump, 0.5μg/kg/min) was started one day before the injection of B16 cells. This dose does not change blood pressure and heart rate in mice.

## Results

When B16/F10 cells (5 x 10^5^ cells) were intravenously injected into C57/B6 mice, 100-150 of visible lung macrometastasis occurred in two weeks. In this hematogenous metastasis model, administration of ANP notably inhibited metastasis in the lung compared to the control mice (13±3 vs. 119±13, P<0.001). Furthermore, lung metastasis significantly increased in the vascular endothelial cell specific GC-A knock-out mice, and decreased in the vascular endothelial cell specific GC-A overexpression mice compared to the wild-type mice. These studies indicate that ANP/GC-A signaling in the vascular endothelial cells plays crucial roles in the inhibition of cancer metastasis.

## Conclusion

We found that ANP inhibits cancer metastasis through GC-A receptor in the vascular endothelial cells. Our data provide novel insights into the prophylactic therapy for various kinds of cancer metastasis.

